# PUF60‐Regulated Isoform Switching of MAZ Modulates Gastric Cancer Cell Migration

**DOI:** 10.1002/cam4.70977

**Published:** 2025-05-24

**Authors:** Dong Xing, Ting Zhao, Chenchen Mao, Zheng Han, Wanxia Cai, Teming Zhang, Dianfeng Mei, Wangkai Xie, Jiaye Yu, Zhonghan Wu, Zhiyuan Chen, Shiyu Feng, Xian Shen, Xiangyang Xue, Dan Xiang

**Affiliations:** ^1^ Wenzhou Collaborative Innovation Center of Gastrointestinal Cancer in Basic Research and Precision Medicine, Wenzhou Key Laboratory of Cancer‐Related Pathogens and Immunity, Department of Microbiology and Immunology, Institute of Molecular Virology and Immunology, School of Basic Medical Sciences Wenzhou Medical University Wenzhou China; ^2^ Department of General Surgery The First Affiliated Hospital of Wenzhou Medical University Wenzhou China; ^3^ Department of General Surgery The Second Affiliated Hospital and Yuying Children's Hospital of Wenzhou Medical University Wenzhou China

**Keywords:** alternative splicing, cell migration, gastric cancer, isoform, Myc‐associated zinc‐finger protein

## Abstract

**Background:**

As an essential transcription factor, Myc‐associated zinc‐finger protein (MAZ) is frequently upregulated in many human tumors and is a well‐documented oncogene. However, we found high expression of MAZ was closely associated with good survival outcomes in patients with stomach adenocarcinoma (STAD), and the underlying mechanism involved remains to be elucidated. We hypothesize that alternative splicing of MAZ plays an important role.

**Methods:**

Pan‐cancer analysis of MAZ expression and prognostic significance was performed using The Cancer Genome Atlas (TCGA) data, with emphasis on its divergent prognostic impact in gastric cancer (GC). MAZ protein levels were further validated in 356 GC tissue samples via immunohistochemistry. Functional investigations encompassed MAZ knockout (KO) and isoform‐specific rescue experiments to assess GC cell migration, alongside quantification of MAZ alternative splicing rates (PSI). Additionally, RNA immunoprecipitation sequencing (RIP‐seq) identified PUF60‐mediated regulation of MAZ isoforms.

**Results:**

MAZ was upregulated in GC but served as an independent protective prognostic factor. MAZ‐KO enhanced GC cell migration, while isoform‐specific re‐expression revealed divergent roles: MAZ‐2 promoted migration, whereas MAZ‐1 and MAZ‐3 suppressed it. Notably, MAZ‐2 is highly expressed in GC and is associated with poor survival prognosis of patients. Lower PSI values of MAZ‐2 were detected in GC. MAZ transcripts were directly bound by PUF60. PUF60 knockdown caused MAZ splice isoform switch, thereby enhancing GC cell migration.

**Conclusion:**

The prognostic difference of MAZ in GC stems from isoform‐specific functional antagonism, with cell migration phenotypes governed by the MAZ‐1/3 versus MAZ‐2 ratio. Targeting MAZ alternative splicing, particularly via PUF60 modulation, represents a novel therapeutic strategy.

## Graphical abstract

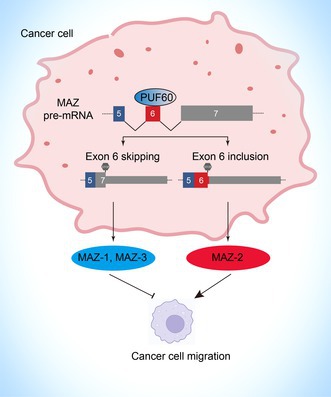

## 1 Introduction

Gastric cancer (GC) is the fifth most common cancer type and the fourth leading cause of cancer death globally [1]. Due to the high molecular and phenotypic heterogeneity of GC, surgical resection is the preferred treatment [2]. However, most patients with advanced GC often have lost the opportunity for surgery. Fortunately, the molecular classification of GC has provided an opportunity for patients to receive personalized treatment. Current targeted agents for GC include trastuzumab to treat HER‐2 positive patients and programmed death receptor‐1 (PD‐1) inhibitor nivolumab to treat patients with microsatellite high instability [2, 3], which have effectively prolonged overall and disease‐free survival. However, these subgroups represent only a small fraction of GC, and novel GC therapeutic targets are urgently needed.

MAZ is a zinc finger protein ubiquitously expressed in various tissues of the human body, and it contains six C2H2 zinc fingers that bind to GA boxes on the promoter of target genes [4]. MAZ has been shown to regulate the expression of a large number of genes, such as proto‐oncogene c‐myc [5], TP53 [6], Ras family gene [7], human telomerase reverse transcriptase (hTERT) [8], vascular endothelial growth factor (VEGF) [9], and cluster of differentiation 4 (CD4) [10], through the dual effects of transcriptional activation and transcriptional termination. Additionally, MAZ is involved in tumor progression by directly binding to the promoter regions of target genes or synergizing with other transcription factors. It has been identified as an oncogene in several tumor types, including hepatocellular carcinoma [11], prostate cancer [12], breast cancer [13], and glioblastoma [14]. There has been evidence that knockdown of MAZ expression inhibits cell proliferation, invasion, migration, and angiogenesis [15]. In addition, MAZ was identified as an insulator‐like factor in genome organization, which is essential for the regulation of global genes, and was an indispensable gene for normal development [16]. These results suggest that MAZ might be a promising therapeutic target in these tumors.

Alternative splicing is an essential mechanism for regulating gene expression in eukaryotes, and more than 95% of human genes cannot be expressed normally without the splicing of pre‐mRNA [17]. In recent years, the abnormal regulation of alternative splicing in cancer initiation and progression has become a tumor marker [18]. Through alternative splicing, a single gene can produce a variety of mature mRNA with entirely different structures and functions, which significantly increases the diversity of proteins. For example, Bcl‐x (L) produced in Bcl‐x pre‐mRNA has anti‐apoptotic ability, while Bcl‐x (S) can promote apoptosis [19]. In addition, alternative splicing of EGFR, KRAS, HRAS, TP53, CD44, and other genes has been reported, which affect almost any stage of tumorigenesis and progression [18, 20, 21, 22, 23]. The MAZ gene encodes three isoforms, MAZ‐1, MAZ‐2, and MAZ‐3, which are highly regulated by alternative splicing [24, 25]. Still, nowadays, most studies on the function of MAZ have focused mainly on MAZ‐1, which has a transcriptional activation function [4]. However, MAZ‐2 could function as a transcriptional repressor of MAZ‐1 [24]. Some studies have found that the expression ratio of the three isoforms of MAZ can be changed in inflammation, suggesting that the functions of the isoforms are different [25]. The functional contribution of MAZ splice isoforms in cancer has not been investigated.

In the present study, we sought to elucidate the expression, clinical correlation, biological function, and potential alternative splicing mechanisms of MAZ in GC. Public databases and clinical samples have confirmed that MAZ expression is upregulated in GC but is associated with improved patient outcomes. We verified that MAZ played an important role in GC metastasis. Among them, MAZ‐2 significantly promoted GC cell migration, while MAZ‐1 and MAZ‐3 had opposite functions. Splicing factor PUF60 is involved in the alternative splicing of MAZ. Our results explain the difference in the prognostic value of MAZ in GC, and the phenotype of GC cell migration can be modulated at the level of the MAZ isoform ratio.

## 2 Materials and Methods

Materials and methods are described in Data S1, including the sequences for sgRNAs and specific primers (Tables S1 and S2).

## 3 Results

### 3.1 Pan‐Cancer MAZ Expression and Associations With Survival

**FIGURE 1:**
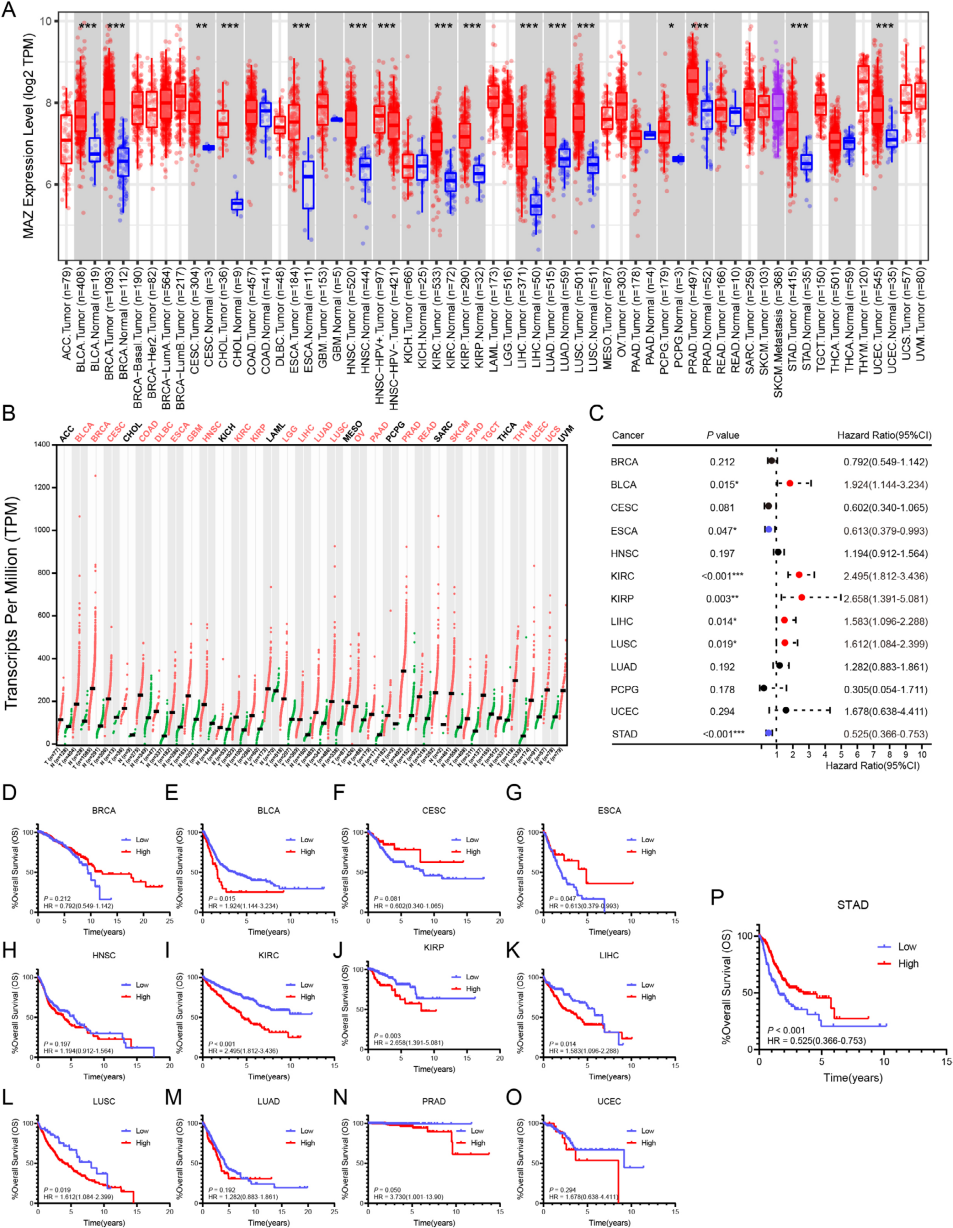
MAZ expression and associations with survival in pan‐cancer. (A) The expression of MAZ from 33 types of cancer samples and corresponding normal samples in the TIMER database. (B) The expression of MAZ from 33 types of cancer samples and corresponding normal samples in the GEPIA database. (C) The correlation between MAZ expression and OS in 13 MAZ‐upregulated tumors. (D–P) Kaplan–Meier analysis of the association between MAZ expression and OS in 13 MAZ‐upregulated tumors.

To evaluate the MAZ expression in pan‐cancer, we first analyzed MAZ mRNA expression between tumor and adjacent normal tissues via the TIMER database [26]. The results showed high MAZ expression in 15 tumor types including BRCA, BLCA, CESC, CHOL, ESCA, HNSC, KIRC, KIRP, LIHC, LUSC, LUAD, PCPG, PRAD, STAD, and UCEC (Figure 1A). Considering the small number of normal tissues in the TCGA database, we used the GEPIA database [27], which integrates TCGA and GTEx data, to further validate the results. MAZ expression was significantly increased in 24 of 33 tumor types, except for ACC, CHOL, KICH, LAML, MESO, PCPG, SARC, THCA, and UVM (Figure 1B). We further explored the relationship between MAZ expression level and prognosis in 13 malignant tumors with up‐regulation of MAZ in both the TIMER and GEPIA databases. Univariate COX regression analysis illustrated MAZ as a high‐risk gene in BLCA, KIRC, KIRP, LIHC, and LUSC, but a low‐risk gene in STAD (*p* < 0.001, HR = 0.525) and ESCA (*p* = 0.047, HR = 0.613) (Figure 1C). As previously reported, MAZ was a cancer‐promoting gene in many malignant tumors [15]. However, high expression of MAZ is most significantly associated with better prognosis in gastric cancer (GC) (Figure 1D–P). To explain the difference in prognosis value of MAZ in GC, we next explored the role of MAZ in GC.

### 3.2 MAZ Upregulation in GC but Correlates With Improved Patient Outcomes

**FIGURE 2:**
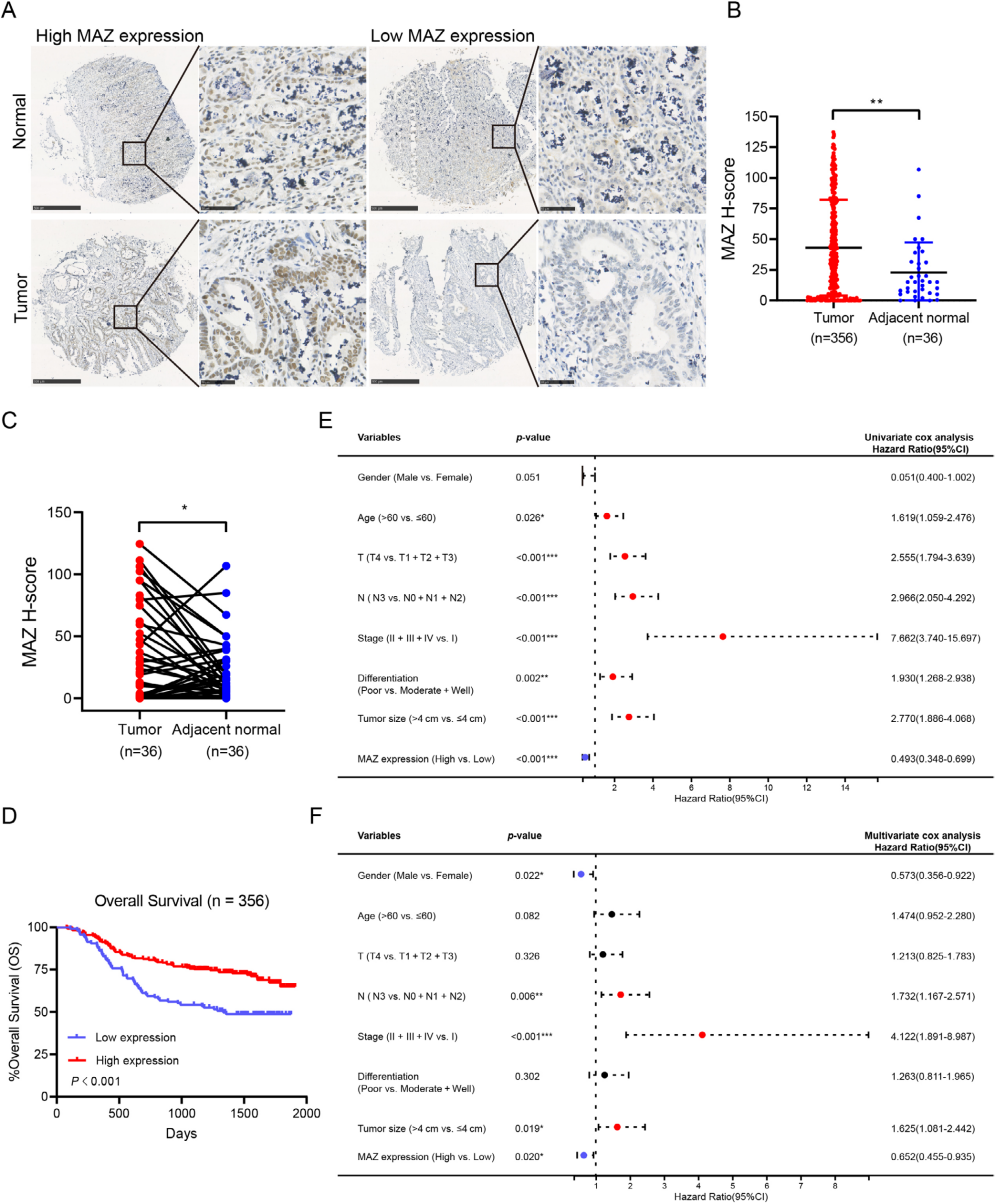
MAZ expression and associations with survival in GC. (A) Representative picture of the IHC staining of MAZ proteins in GC and normal tissues. Scale bar, 500 μm (low magnification) or 50 μm (high magnification). (B) Compared MAZ H‐scores in GC tissues (*n* = 356) and adjacent normal tissues (*n* = 36). (C) Compared MAZ H‐scores in paired GC tissues and adjacent normal tissues (*n* = 36). (D) Kaplan–Meier curve showed MAZ gene expression predicted better OS (*n* = 356, *p* < 0.001) in patients with GC. (E, F) Univariate and multivariate Cox regression analysis of OS in patients with GC.

We detected the expression of the MAZ gene by immunohistochemistry (IHC) in our archived GC tissue microarray and found that MAZ was expressed mainly in the nucleus of cancer cells (Figure 2A). To analyze the expression of MAZ in detail, the IHC results were scored by the H‐score method. MAZ was highly expressed in tumor tissues compared with adjacent normal tissues (Figure 2B,C). According to the different H‐scores, patients with GC were divided into the MAZ high expression group (*n* = 240) and the MAZ low expression group (*n* = 116). It was shown that patients with MAZ high expression had better T‐stage (*p* = 0.026), better TNM stage (*p* < 0.001), and smaller tumor size (*p* = 0.004) than those with MAZ low expression; no significant correlations were observed between MAZ and gender, age, N stage, or differentiation (*p* > 0.05) (Table 1). Kaplan–Meier survival analysis showed that patients with MAZ high expression had more prolonged overall survival (OS) than those with low MAZ expression (Figure 2D). Moreover, multivariate analysis revealed that high expression of MAZ was an independent protective predictor of OS (Figure 2E,F). These results indicate that MAZ upregulation in GC is associated with better prognosis in patients with GC.

**TABLE 1:** Clinicopathological features and MAZ in GC.

Variables	MAZ expression		
	Low (*n* = 116, 32.6%)	High (*n* = 240, 67.4%)	*p*
**Gender (*n* [%])**			0.664
Male	87 (75.0%)	185 (77.1%)	
Female	29 (25.0%)	55 (22.9%)	
**Age (*n* [%])**			0.690
< 60	31 (26.7%)	69 (28.8%)	
≥ 60	85 (73.3%)	171 (74.2%)	
**Depth of invasion (*n* [%])**			0.026*
T1 + T2 + T3	60 (52.2%)	153 (64.6%)	
T4	55 (47.8%)	84 (35.4%)	
**Lymph node metastasis (*n* [%])**			0.218
N0 + N1 + N2	88 (76.5%)	197 (82.1%)	
N3	27 (23.5%)	43 (17.9%)	
**TNM stage (*n* [%])**			< 0.001***
I	15 (13.2%)	83 (35.0%)	
II + III + IV	99 (86.8%)	154 (65.0%)	
**Differentiation status (*n* [%])**			0.153
Well and moderate	30 (25.9%)	80 (33.3%)	
Poor	86 (74.1%)	160 (66.7%)	
**Tumor size (cm) (*n* [%])**			0.004**
< 4	42 (36.5%)	126 (52.9%)	
≥ 4	73 (63.5%)	112 (47.1%)	

* *p* < 0.05.

** *p* < 0.01.

*** *p* < 0.001.

### 3.3 MAZ Inhibits the Migration of GC Cells

**FIGURE 3:**
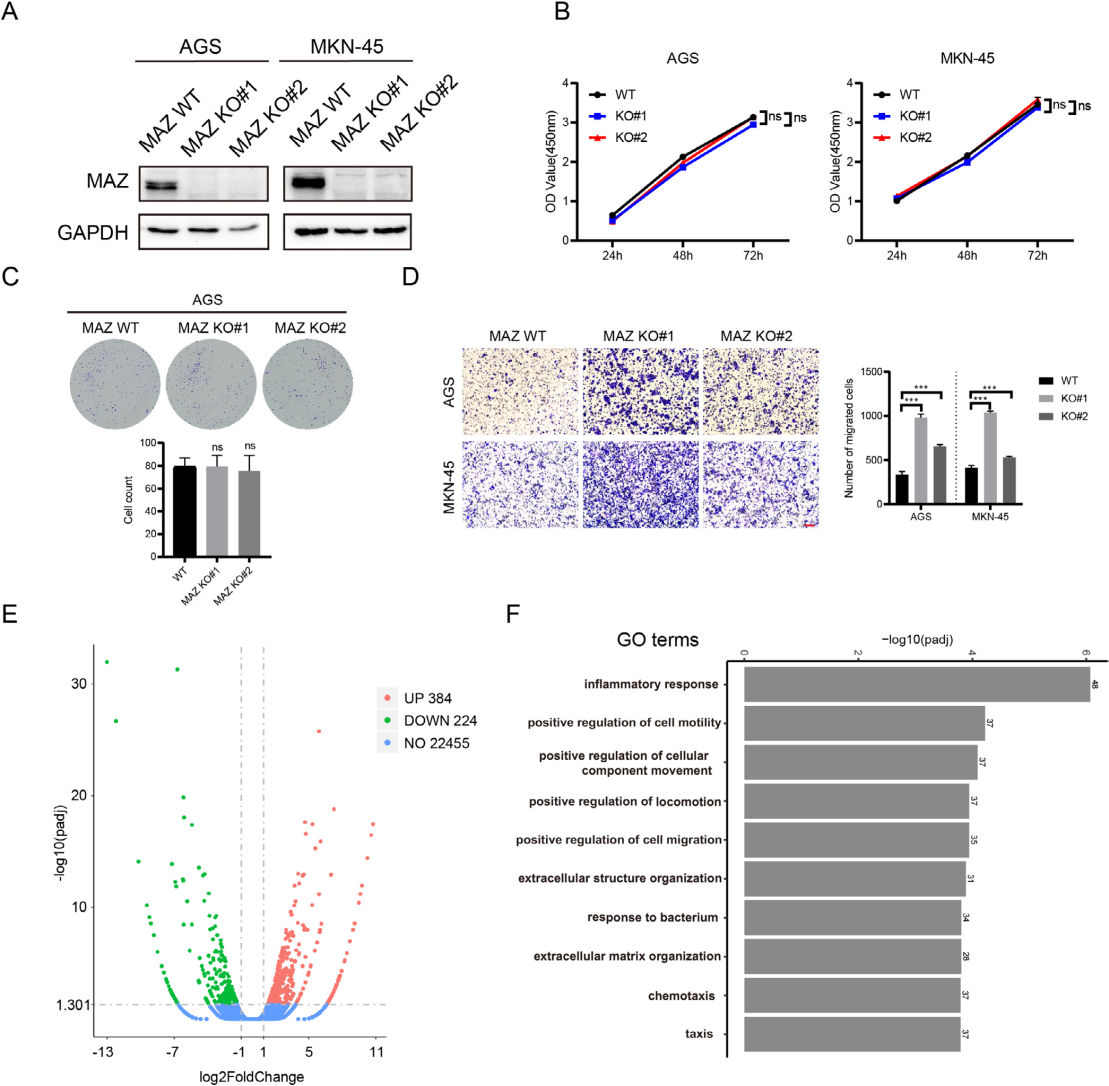
MAZ acts as a suppressor of cancer cell migration in GC progression. (A) Western blot analysis of MAZ knockout in AGS and MKN‐45 cells. (B, C) The proliferation of cells under MAZ knockout was determined via CCK‐8 assay and colony formation assay, respectively. (D) The migration of cells under MAZ knockout was determined via transwell assay. Scale bar, 100 μm. (E) RNA‐seq in MAZ knockout AGS and control cells. Volcano plot showing 608 DEGs. (F) GO enrichment of DEGs between MAZ knockout AGS and control cells.

To explore how MAZ might affect GC progression, we generated heterozygous MAZ knockout AGS and MKN‐45 GC cells (MAZ+/−) by CRISPR/Cas9. Individual clone populations were screened for MAZ expression by western blot, and two independent clones without MAZ expression were obtained in AGS and MKN‐45, respectively (Figure 3A and Figure S1A). We observed that the growth rates of MAZ knockout cells (MAZ‐KO) and wide‐type cells (MAZ‐WT) were almost equal (Figure 3B,C and Figure S1B). Although there was heterogeneity in cell migration between the two independent clones, MAZ‐deficient cells showed significantly enhanced migration compared with MAZ‐WT cells (Figure 3D). To examine the biological processes altered by MAZ, we performed RNA sequencing (RNA‐seq) in MAZ‐knockout AGS cells. As a result, a total of 608 differentially expressed genes (DEGs) were screened out, of which 384 DEGs were upregulated and 224 DEGs were down‐regulated (Figure 3E). Gene ontology (GO) enrichment showed that in addition to being related to “immune response”, these DEGs were highly enriched for terms related to cell migration, such as “positive regulation of cell motility”, “positive regulation of cellular component movement”, “positive regulation of locomotion”, “positive regulation of cell migration”, “extracellular structure organization”, “extracellular matrix organization and chemotaxis” (Figure 3F), and MAZ has been shown to regulate the production of pro‐tumor cytokines in LUAD, playing an important role in immune evasion [28]. Overall, this evidence above supports the inference that MAZ inhibits GC cell migration.

### 3.4 MAZ Isoforms Displayed Opposite Roles in GC Cell Migration

**FIGURE 4:**
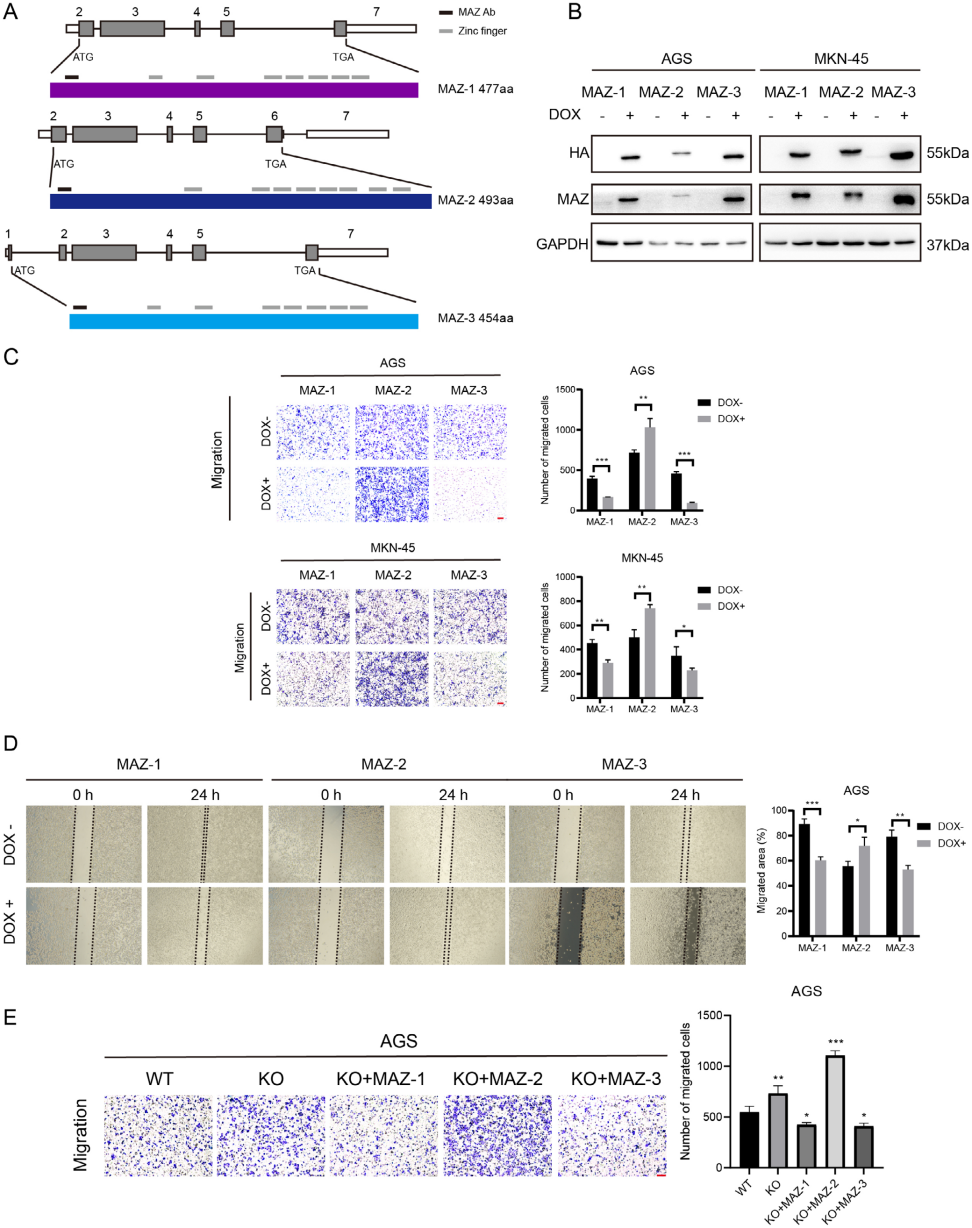
MAZ‐1 and MAZ‐3 suppress GC cell migration while MAZ‐2 enhances GC cell migration. (A) Schematic of MAZ pre‐mRNA and MAZ splice isoforms. MAZ antibody‐targeting regions and zinc finger domains are indicated. (B) Western blot analysis of MAZ of AGS and MKN‐45 cells expressing MAZ‐1‐HA, MAZ‐2‐HA, or MAZ‐3‐HA. (C) Representative images from transwell assays with AGS and MKN‐45 cells expressing MAZ‐1‐HA, MAZ‐2‐HA, or MAZ‐3‐HA. Scale bar, 100 μm. (D) Representative images from wound healing assays with AGS cells expressing MAZ‐1‐HA, MAZ‐2‐HA, or MAZ‐3‐HA. (E) Representative images from transwell assays with AGS MAZ‐KO cells re‐expressing MAZ‐1‐HA, MAZ‐2‐HA, or MAZ‐3‐HA. Scale bar, 100 μm.

In the MAZ gene family, three members that have been identified are formed by alternative splicing (Figure 4A). Compared with MAZ‐1, MAZ‐2 is generated due to the retention of exon 6, and the difference in the carboxyl terminus confers two additional zinc fingers [24]. In addition, MAZ‐3 with different N termini was generated due to the different transcription start sites [25]. The structural differences of MAZ isoforms lead to different transcriptional activities [25]. We generated MAZ stable transfected GC cell lines AGS and MKN‐45 based on the tet‐on system, which could express MAZ‐1, MAZ‐2, and MAZ‐3 after doxycycline induction, respectively, and a HA tag was added to the C‐terminus of MAZ. Western blotting and immunofluorescence (IF) staining detected the overexpression of the MAZ isoform (Figure 4B and Figure S1C,D). Next, transwell assay and wound healing assay were performed to explore whether MAZ isoforms have an equal effect on cell migration. Notably, MAZ‐1 and MAZ‐3 overexpression significantly inhibited the migration of both GC cells, but overexpression of MAZ‐2 resulted in a marked increase in cell migration (Figure 4C,D). Western blot results showed that overexpression of MAZ‐1 led to decreased expression levels of Vimentin and N‐cadherin, while overexpression of MAZ‐2 promoted the expression of Vimentin and N‐cadherin (Figure S1E). Moreover, we reintroduced the gene into AGS MAZ KO#1 cells, and the transwell assay showed similar results. MAZ‐1 and MAZ‐3 re‐expression rescued the increased migration induced by MAZ‐KO. Conversely, the re‐expression of MAZ‐2 further enhanced GC cell migration (Figure 4E).

### 3.5 MAZ Isoform Ratio Is Associated With Patient Survival

**FIGURE 5:**
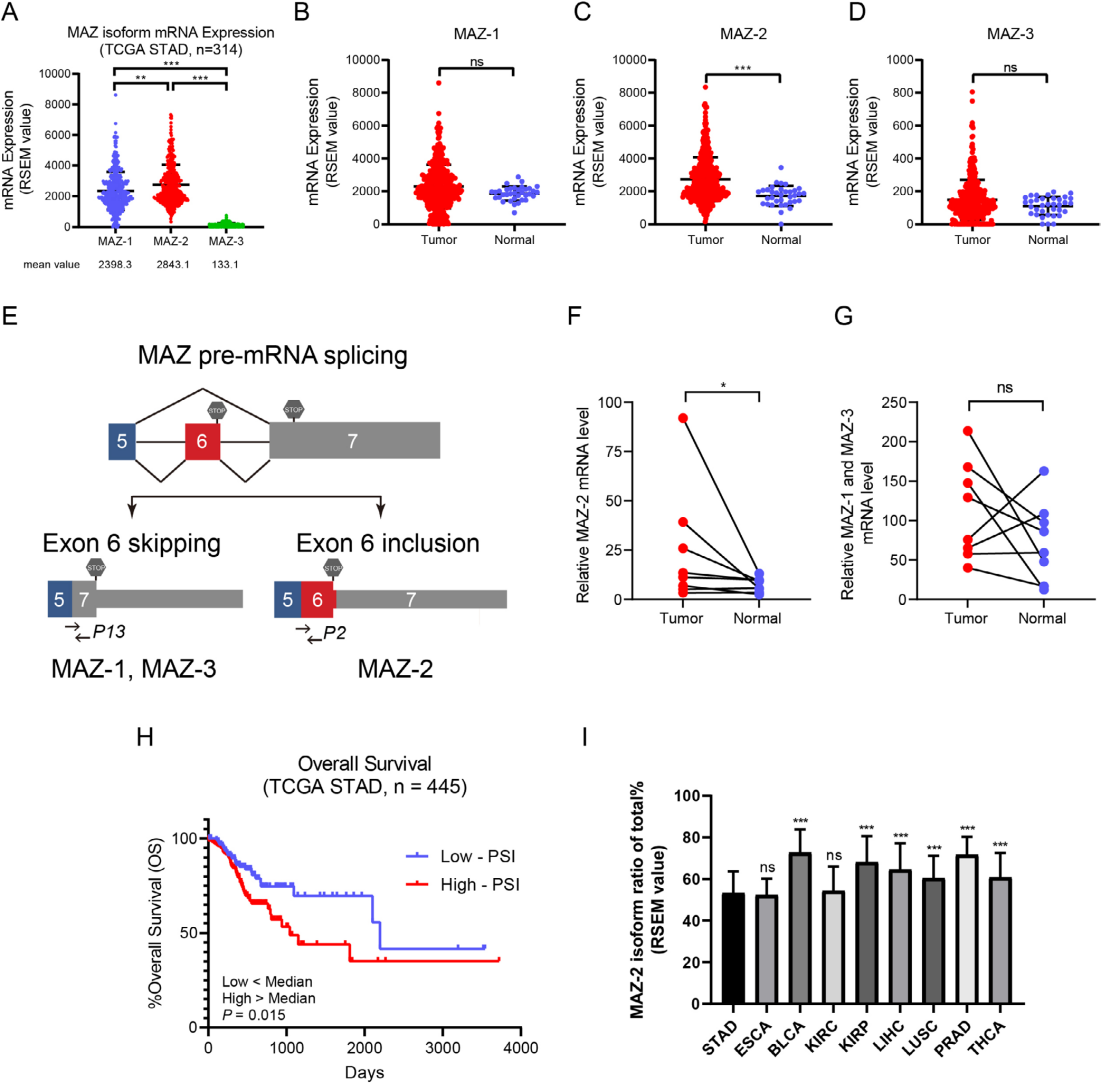
MAZ isoform ratio is associated with patient survival. (A–D) MAZ isoform mRNA levels in STAD patients derived from TCGA data. (E) Schematic of MAZ pre‐mRNA splicing. The positions of primers used in the RT‐PCR analysis of MAZ isoforms are indicated. (F, G) The expression levels of MAZ isoforms in 8 paired GC tissues and adjacent normal tissues were detected by RT‐PCR. (H) Kaplan–Meier curves for overall survival in STAD and MAZ PSI values from the TCGASpliceSeq. The patients with high‐PSI had a worse survival outcome (*n* = 445, *p* = 0.015). PSI = splice‐in / (splice‐in + splice‐out). (I) MAZ‐2 isoform ratio of total is low in STAD and ESCA.

Given the different effects of MAZ isoforms on GC cell migration, we investigated the distribution of their transcripts via the publicly available TCGA Splice Variant database (TSVdb) [29]. We found that in 314 GC patients, the abundance of MAZ‐2 mRNA was slightly higher than that of MAZ‐1 mRNA. In contrast, the abundance of MAZ‐3 mRNA was very low (Figure 5A). In addition, we found that MAZ‐2 mRNA, which promotes tumor cell migration, was more abundant in GC tissues, while the abundance of MAZ‐1 and MAZ‐3 mRNA was not statistically different between tumor and adjacent normal tissues (Figure 5B–D). Because both MAZ‐1 and MAZ‐3 contributed to cell migration inhibition, we designed specific primers for the additional exon 6 of MAZ‐2, as shown in Figure 5E, and then examined the abundance of each MAZ transcript in eight paired pairs of GC tissues. Similar to these results obtained from the TCGA database, the mRNA level of MAZ‐2 was significantly higher in GC tissues than in the adjacent tissues (Figure 5F,G). These findings led us to examine whether MAZ exon 6 skipping is associated with prognosis in patients with GC. We divided 445 GC patients in TCGA into percent‐splice‐in (PSI)‐high and PSI‐low groups based on the exon 6 skipping event, and patients in the PSI‐high group had a worse survival outcome (Figure 5H). Taken together, we postulated that the differential effects of MAZ in GC prognosis might be related to diversity in MAZ isoform expression. Interestingly, except for KIRC, the MAZ‐2 isoform ratio in BLCA, KIRP, LIHC, and LUSC was significantly higher than that in STAD and ESCA. At the same time, MAZ was a protective factor for prognosis in STAD and ESCA and a risk factor for prognosis in BLCA, KIRP, LIHC, and LUSC. These results indicate that the MAZ isoform ratio is associated with patient survival (Figure 5I).

### 3.6 Splicing Factor PUF60 Regulates Alternative Splicing of MAZ

**FIGURE 6:**
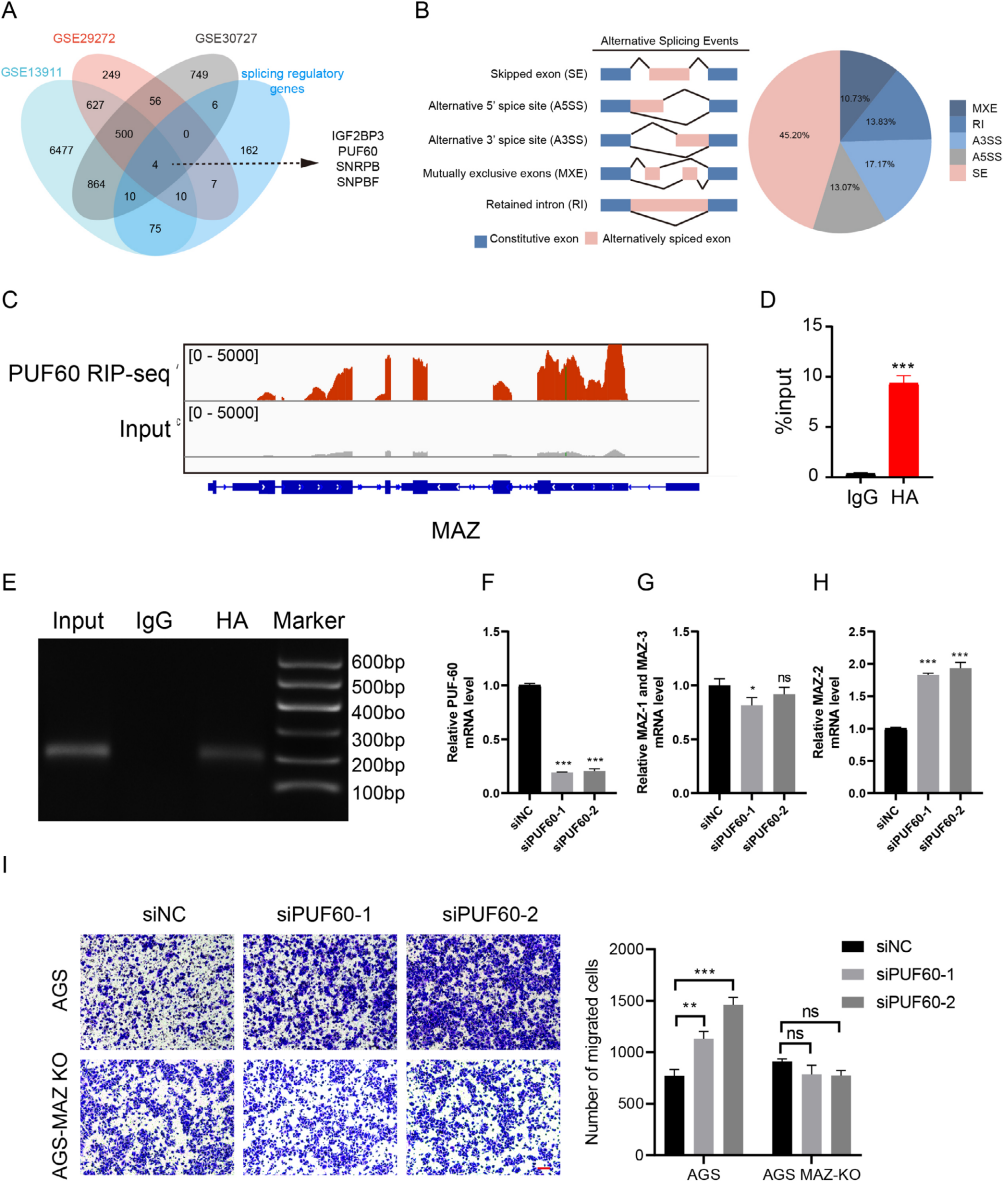
Splicing factor PUF60 regulates alternative splicing of MAZ. (A) Co‐differentially expressed splicing regulatory gene in GSE13911, GSE29272, and GSE30727 datasets. (B) PUF60‐regulated AS events in AGS cells. The classification of alternative splicing events includes skipped exon (SE), alternative 5ʹ splice site (A5SS), alternative 3ʹ splice site (A3SS), mutually exclusive exon (MXE), and retained intron (RI). (C) Peak analysis identified RIP‐seq peaks on MAZ exons enriched by PUF60, which were shown as track signals in an integrative genomic viewer (hg38). (D, E) RIP‐PCR analysis of PUF60 binding sites. Isotype antibody was used as the control. (F–H) The expression levels of MAZ isoforms in AGS cells with PUF60 knockdown were detected by RT‐PCR. I Representative images from transwell assays with knockdown of PUF60 in MAZ‐KO AGS or WT‐AGS cells. Scale bar, 100 μm.

To explore the underlying mechanism of MAZ alternative splicing in GC, a total of 504 co‐DEGs were obtained by using the GEO2R from GSE13911, GSE29272, and GSE30727 datasets. In addition, we examined the expression levels of splicing regulatory genes that have been reported [30]. Finally, we obtained four differentially splicing‐regulated genes in GC (Figure 6A), which were IGF2BP3, PUF60, SNRPB, and SNRPF. Additionally, our RNA‐seq results suggested that overexpression of PUF60 in AGS cells resulted in 21,234 splicing events, including 9838 skipped exons, 2232 mutually exclusive exons, 2718 alternative 5′ splice sites, 3571 alternative 3′ splice sites, and 2875 retained introns, and PUF60 is involved in exon skipping in MAZ (Figure 6B). Therefore, the splicing factor PUF60 was selected for further investigation. RNA immunoprecipitation sequencing (RIP‐Seq) assays were performed on AGS cells overexpressing PUF60‐HA with antibodies against HA‐tag to evaluate the accuracy of our RNA‐seq analysis in PUF60 splicing changes. As expected, PUF60 binding peaks are observed on each exon of the MAZ transcripts (Figure 6C). Moreover, RIP‐PCR was conducted and confirmed that MAZ transcripts were directly bound by PUF60 (Figure 6D,E).

To explore the role of PUF60 in MAZ alternative splicing, we examined the expression of each MAZ isoform in PUF60‐silenced conditions. PUF60 knockdown led to increased expression of MAZ‐2 but decreased expression of MAZ‐1 and MAZ‐3 slightly (Figure 6F–H). As an essential splicing factor involved in the progression of various kinds of tumors [31], we speculated that PUF60 regulates the migratory phenotype of GC cells through alternative splicing of MAZ. Consistent with this hypothesis, the migration of GC cells was enhanced after knocking down PUF60 (Figure 6I). Notably, when PUF60 was knocked down in MAZ‐KO cell lines, there was no significant change in cell migration (Figure 6I).

In this study, we found that the MAZ‐2 isoform possessed potent pro‐tumor metastatic activity, whereas MAZ‐1 and MAZ‐3 have opposite effects in GC. The splicing factor PUF60 regulates alternative splicing of MAZ, and the phenotype of cell migration can be modulated at the level of MAZ alternative splicing. Our finding unravels that targeting MAZ alternative splicing is a potential therapeutic approach.

## 4 Discussion

Gastric cancer (GC) is one of the most common malignant gastrointestinal tumors, and metastasis is the deadliest step in the progression of GC. Even after radical surgery, patients with advanced GC still face metastasis and recurrence, which often means a terrible outcome [32]. The factors involved in metastasis are very complex and multifaceted and are still not fully understood, making it difficult to inhibit the metastasis of GC effectively. Previous studies have detected that the activity of transcription factors (TFs) regulating normal stomach and GC has been implicated in GC invasiveness [33, 34]. New studies have also shown that activation of a small set of TFs driving the mesenchymal phenotype plays an important role in GC progression [34, 35]. Targeting TFs may represent potential therapeutic opportunities. This study is the first to systematically investigate the relationship between myc‐associated zinc‐finger protein (MAZ) isoforms and tumor metastasis, as well as the clinical and prognostic value in GC. Our results demonstrate that the prognosis suggested by MAZ is specific in GC across all TCGA tumors. MAZ, a member of the C2H2 transcription factor family, is involved in gene expression and tumor development. In prostate cancer, MAZ promotes bone metastasis through transcriptionally activating the KRas‐dependent RalGEFs pathway, and MAZ binds to the CDH1 promoter to promote epithelial‐mesenchymal transition (EMT) [12, 36]. In glioblastoma, hepatocellular carcinoma, and neuroblastoma, MAZ is associated with the promotion of tumor cell migration and invasion [15]. However, in basal‐like breast cancer, the MAZ‐FOXF2 axis has a dual function of promoting proliferation and inhibiting migration [37], and MAZ did not significantly impact migration and invasion capabilities in papillary thyroid carcinoma [38]. Studies have shown that the knockdown of MAZ under acidic stimulation inhibits the EMT process in GC, thereby inhibiting GC cell migration [37]. On the contrary, our study shows that MAZ knockdown in GC cells significantly enhanced cell migration. Moreover, in our cohort of GC patients, we demonstrated that MAZ was highly expressed in GC tissues compared to adjacent normal tissues, but high expression of MAZ is associated with more prolonged overall survival of patients. All these contradictory findings suggest that the effect of MAZ on tumor metastasis is more complex than previously thought.

In this study, we demonstrated that MAZ splice isoforms exhibit distinct activities in GC migration; MAZ‐1 and MAZ‐3 inhibit GC cell migration, while MAZ‐2 exerts the opposite functions (Figure 4C–E and Figure S1E). Until now, little is known about the function of MAZ‐2. Due to alternative splicing of the precursor mRNA, exon 6, which contains 225 nucleotides, is retained at the C‐terminus of MAZ‐2 mRNA. This splicing event causes the stop codon to appear prematurely, making the difference in the MAZ‐2 structure. Compared with MAZ‐1 and MAZ‐3, MAZ‐2 has two additional zinc finger structures at the C‐terminus, which makes MAZ‐2 have different transcriptional activities [24]. However, whether this is the reason why MAZ‐2 promotes GC cell migration needs to be further explored. We found that MAZ‐2 was highly expressed in GC tissues compared to adjacent normal tissues, but there was no significant difference between MAZ‐1 and MAZ‐3. Furthermore, the MAZ isoform ratio is associated with patient survival. Our results showed that the MAZ‐2 isoform ratio in STAD and ECSA (prognostic protective factors) was significantly lower than that in BLCA, KIRP, LIHC, and LUSC (prognostic risk factors) (Figure 5I). Interestingly, we also found that the MAZ‐2 isoform ratios of the total in GC (pan‐MAZ inhibited cell migration), papillary thyroid carcinoma (pan‐MAZ did not affect cell migration), and hepatocellular carcinoma (pan‐MAZ promoted cell migration) were 53.27%, 60.76%, and 64.54%, respectively (Figure 5I). These results indicate that the MAZ isoform ratio regulates cancer cell migration and is associated with patient prognosis.

To target MAZ alternative splicing, we screened PUF60, a splicing factor with significantly altered expression in GC. PUF60 is an RNA‐binding protein that has been identified as a component of the spliceosome and plays a key role in pre‐mRNA splicing [39]. Our RNA‐seq and RIP‐seq data revealed that PUF60 is a critical splicing regulator of MAZ; PUF60 could bind directly to MAZ transcripts. Recently, PUF60 has been reported to be involved in promoting cell cycle and lung cancer progression by regulating alternative splicing of CDC25C [40]. In addition, PUF60 is closely related to c‐myc transcription and tumor processing [40, 41]. Interestingly, c‐myc was one of the first identified and most important target genes of MAZ [4]. We demonstrated that the mRNA abundance of MAZ‐2 was significantly upregulated under PUF60 knockdown conditions while promoting GC cell migration (Figure 6F–H). However, the reduced PUF60 level had no significant effect on migration when MAZ was knocked out (Figure 6I). Therefore, these results suggest that PUF60 regulates GC cell migration by affecting MAZ alternative splicing. PUF60 and MAZ together play a critical role in the regulation of tumor progression.

In recent years, a variety of approaches targeting alternative splicing have been under clinical development, including inhibition of key splicing factors or modulation of specific AS events [42]. Our study demonstrated the opposing functions of MAZ isoforms in GC migration. MAZ‐2, which has the potential to increase migration, is highly expressed in tumors. We also provided evidence that the splicing factor PUF60 binds directly to MAZ transcripts. Notably, MAZ isoform ratios are altered during PUF60 knockdown. Using small molecules, antisense oligonucleotides, CRISPR‐based approaches or engineered small nuclear RNAs to target MAZ splice‐switching may be potential options for inhibiting metastasis in GC.

## Author Contributions

**Dong Xing:** investigation (equal), validation (equal), writing – original draft (equal). **Ting Zhao:** investigation (equal), validation (equal), writing – original draft (equal), writing – review and editing (equal). **Chenchen Mao:** data curation (equal), software (equal). **Zheng Han:** writing – review and editing (equal). **Teming Zhang:** investigation (equal), methodology (equal). **Dianfeng Mei:** investigation (equal), validation (equal). **Wangkai Xie:** investigation (equal), methodology (equal). **Jiaye Yu:** resources (equal). **Zhonghan Wu:** resources (equal). **Zhiyuan Chen:** resources (equal). **Shiyu Feng:** resources (equal). **Xian Shen:** conceptualization (equal), funding acquisition (equal), resources (equal), supervision (equal). **Xiangyang Xue:** conceptualization (equal), funding acquisition (equal), project administration (equal), resources (equal), supervision (equal). **Dan Xiang:** data curation (equal), project administration (equal), software (equal), supervision (equal). **Wanxia Cai:** methodology (equal).

## Ethics Statement

All studies were approved by the Ethics Committee of the First Affiliated Hospital of Wenzhou Medical University (LCKY2020‐43). All patients provided written informed consent to participate in the study.

## Conflicts of Interest

The authors declare no conflicts of interest.

## Supporting information


**Data S1.** Supporting Information.

**Figure S1.** (A) Genomic sequencing analysis of AGS partial knockout cells. (B) The proliferation of cells under MAZ knockout was determined via EdU assays. Scale bar, 100 μm. (C, D) Western blot analysis and immunofluorescence to detect MAZ splice isoform localization in AGS cells expressing MAZ‐1‐HA, MAZ‐2‐HA, or MAZ‐3‐HA. Scale bar, 25 μm. (E) Western blot analysis to the expression of EMT markers in AGS cells expressing MAZ‐1‐HA, and MAZ‐2‐HA.

**Table S1.** Sequences of gRNA and primers.

**Table S2.** GO enrichment analysis of AGS MAZ KO cells.

## Data Availability

All data generated or analyzed during this study are included in this published article.
