# Supplementary material for: PUF60‐Regulated Isoform Switching of MAZ Modulates Gastric Cancer Cell Migration
Source: Cancer Med. 2025 May 24;14(11):e70977. doi: 10.1002/cam4.70977 (PMC12102611; doi:10.1002/cam4.70977)
Supplement: Supplementary file 1 — Data S1. Supporting Information. [file CAM4-14-e70977-s004.docx]

**RNA-seq and analysis**

MAZ mRNA expression profiles in 33 human tumors and normal tissues from the TCGA database and GTEx were analyzed by TIMER2.0 (http://timer.cistrome.org) and GEPIA (http://gepia.cancer-pku.cn). The clinical data of 13 types of tumor tissues were sourced from TCGA (http://cancergenome.nih.gov) to analyze the correlation between patient outcomes and MAZ mRNA expression. The PSI (Percent-Spliced-In) of AS events and MAZ isoforms expression were downloaded from TCGA SpliceSeq (https://bioinformatics.mdanderson.org/TCGASpliceSeq/index.jsp) and TCGA Splice Variant database (TSVdb) (http://tsvdb.com), respectively. To find differentially expressed genes (DEGs) between GC tissues and normal tissues, GSE13911, GSE29272, and GSE30727 datasets were downloaded from Gene Expression Omnibus (https://www.ncbi.nlm.nih.gov/geo) and analyzed by GEO2R.

For RNA-seq of GC cells, after total RNA extraction from AGS cells with MAZ knockdown or PUF60 overexpression, RNA-seq was performed based on the Illumina system according to the manufacturer’s instructions. Differential expression analysis between experimental and control groups was performed using the DESeq2 R package. The threshold of DEGs was |log2FC| > 1 and *P* < 0.05. GO enrichment analysis of DEGs was performed using clusterProfiler software. AS event analysis between experimental and control groups was performed using the rMATS software.

**Patients and specimens**

After excluding patients with incomplete histopathological tissue, 356 patients who underwent gastrectomy (in whole or in part) and were pathologically verified as gastric cancer at the First Affiliated Hospital of Wenzhou Medical University (Wenzhou, China) from January 2014 to December 2016 were enrolled. We obtained GC and paired adjacent normal tissue specimens embedded in paraffin and collected essential clinical features, including the age, gender, tumor size, differentiation status, and TNM staging. Subsequently, we constructed a tissue microarray (TMA). All studies were approved by the Ethics Committee of the First Affiliated Hospital of Wenzhou Medical University. All patients provided written informed consent to participate in the study.

**Cells culture**

The GC cell line AGS was obtained from American type culture collection (ATCC, Manassas, VA, USA) and cultured in RPMI-1640 medium supplemented with 10% fetal bovine serum (FBS). The GC cell lines MKN-45 were obtained from the Chinese Academy of Sciences (Shanghai Institute of Biochemistry and Cell Biology, Shanghai, China) and cultured in Dulbecco's Modified Eagle Medium (DMEM) supplemented with 10% FBS. All cells were cultured at 37 °C in a humidified 5% CO_2_ atmosphere. All cell lines were tested negative for mycoplasma contamination.

**MAZ knockout cell lines generation**

To generate MAZ knockout GC cell lines, the sgMAZ were designed (TableS2). After annealing, the DNA oligos were inserted into PX459 (pSpCas9(BB)-2A-Puro) plasmid. Then, the recombinant plasmids were transfected into AGS and MKN-45 cells using lipofectamine 2000 reagent (Thermo Fisher, CA, USA). GC cells were screened by puromycin (Invitrogen; 2 µg/mL) for one week.

**Stable cell lines generation**

To establish stable transfected cells, the full-length sequences of MAZ isoforms 1, 2, and 3, with the C-terminal HA-tag were cloned into the pInducer20 vectors (RRID: Addgene_44012). Lentivirus particles were produced in HEK293T cells and infected the GC cells using the Lenti-X HTX packaging system (Takara, Tokyo, Japan). Infected GC cells were screened by G418 (Invitrogen) for one week. Doxycycline (DOX; Sigma‐Aldrich, St. Louis, Missouri, USA; 1 μg/mL) was used to induce MAZ isoforms expression, which was inserted into the tetracycline response promoter TET-on system.

**Immunohistochemistry**

TMAs were constructed as described previously and MAZ gene expression was detected using immunohistochemistry. Firstly, TMAs were dewaxed at 60 °C for 1 h, then hydrated with gradient alcohol using sodium citrate buffer solution (Zhongshan Golden Bridge Biotechnology, Beijing, China) for antigen recovery, cultured in hydrogen peroxide solution for 10 min to eliminate endogenous catalase, washed with PBS, blocked with goat serum for 30 min, incubated with MAZ antibody at 25 °C for 2 h in a wet box. Then the slides were incubated with 3,3'-diaminobenzidine (Dako, Carpinteria, CA, USA) at 25 °C for 30 min and, finally, re-dyed with hematoxylin, hydrated with gradient alcohol, and sealed with neutral gum.

**Western blot analysis**

The total proteins of GC cells were extracted at 4 °C using RIPA with protease inhibitor (Beyotime Institute of Biotechnology, Beijing, China). The total protein concentration was measured using a BCA kit (Beyotime Institute of Biotechnology). Samples were separated using SDS-PAGE and then electrophoretically transferred to polyvinylidene fluoride membranes (Millipore). Membranes were blocked with 5% non-fat milk for 1 h at 25 °C, then washed with PBST and incubated with antibodies overnight at 4 °C. The following antibodies were used: MAZ (Proteintech, #21068-1-AP, 1:1000), HA-tag (CST, #3274, 1:1000) and GAPDH (Goodhere, Hangzhou, China, AB-P-R, 1:1000). After washing the membranes three times with TBST, membranes were incubated with HRP-conjugated antibodies (Cell signal Technology, 1:1000) for 1 h at 25 °C. Proteins were detected using an ECL system.

**Immunofluorescence**

The GC cells were inoculated and adhered to aseptic cover slides. After induction of MAZ expression by DOX, the slides were fixed in 4% paraformaldehyde for 10 min at 25 °C, and 0.1% TritonX-100 was infiltrated for 10 min at 37 °C. The cells were then stained with MAZ (Proteintech, #21068-1-AP, 1:500) or HA-tag (CST, 1:1000) antibodies. FITC-conjugated goat anti-mouse or anti-rabbit IgG was used as the secondary antibody (Invitrogen). Nuclei was stained with DAPI (Beyotime Institute of Biotechnology). Signals were observed and recorded using a fluorescence microscope (Nikon Cl-I, Japan).

**Transwell migration assay**

A chamber with a pore diameter of 8 μm (Corning, USA) was used to evaluate the migration abilities of GC cells. After induction of MAZ expression by DOX, the cells were suspended in the upper chamber with 200 μL serum-free DMEM, and 500 μL DMEM containing 10%FBS was added to the lower cavity. After being cultured at 37 °C for 12 h, Transwell inserts were washed with PBS, fixed with 4% paraformaldehyde for 15 min, and stained with 0.1% crystal violet for 15 min. The results were observed using an inverted optical microscope.

**Wound healing assay**

For wound healing assays, starved MAZ-stable AGS cells (4 × 10^5^ cells/well) were seeded into 6-well plates. After DOX induction, the wound was made with sterile 200 μL pipettor tips in a monolayer of cells. Wound was imaged at the same location at 0h and 24h using microscope.

**Cell viability**

Cell count kit-8 (CCK-8) (Dojindo, Japan) was used to evaluate cell viability. GC cells were cultured in 96-well plates (5 × 10^3^ cells/well). After incubation at 37 °C for 24 h or 48 h with 5% CO_2_, cells were treated with a CCK-8 reagent at 37 °C for 2 h. Absorbance was measured at 450 nm using a microplate reader. All experiments were carried out in triplicate.

**Colony formation assay**

GC cells were cultured in 6-well plates (500 cells/well). After incubation at 37 °C for one week with 5% CO_2_, cells were washed with PBS and then fixed with 4% polymethanol for 20 min, and stained with 0.1% crystal violet for 20 min. The results were observed using an inverted optical microscope.

**RNA** **immunoprecipitation assay**

RNA immunoprecipitation (RIP) assays were performed using the EZ-Magna RIP™ RNA-Binding Protein Immunoprecipitation Kit (Sigma-Aldrich, #17-701) following the manufacturer’s instructions. In brief, after pcDNA3.1/PUF60-HA vector transfection for 48 h, AGS cells were collected using cell scratchers and suspended in RIP lysis buffer, and 10% of the lysates were collected as input. For immunoprecipitation, HA-tag antibodies or IgG isotype control were then added to the cell lysate and incubated on a shaker at 25 °C for 0.5 h. After washed twice with lysis buffer, the magnetic bead- antibody complex obtained, and RNA was isolated for RT-PCR analysis.

Statistical analysis

The chi-square test and Fisher exact test were used for classified variables. Student’s t-test was used for continuous variables that follow the normal distribution, and Mann-Whitney U-test was used for continuous variables that did not follow the normal distribution. Overall survival rate (OS) was estimated using Kaplan-Meier analysis and logarithmic rank test. One-way or two-way ANOVA were performed for multiple comparisons. All statistical analyses were conducted using R and SPSS V23 (RRID: SCR_002865) (Chicago, Illinois, USA) for Windows. A value of *P* < .05 was considered statistically significant. The significance levels are labeled as follows: * *P* < .05; ** *P* < .01; and *** *P* < .001.
