# Supplementary figures and images for: PUF60‐Regulated Isoform Switching of MAZ Modulates Gastric Cancer Cell Migration
Source: Cancer Med. 2025 May 24;14(11):e70977. doi: 10.1002/cam4.70977 (PMC12102611; doi:10.1002/cam4.70977)

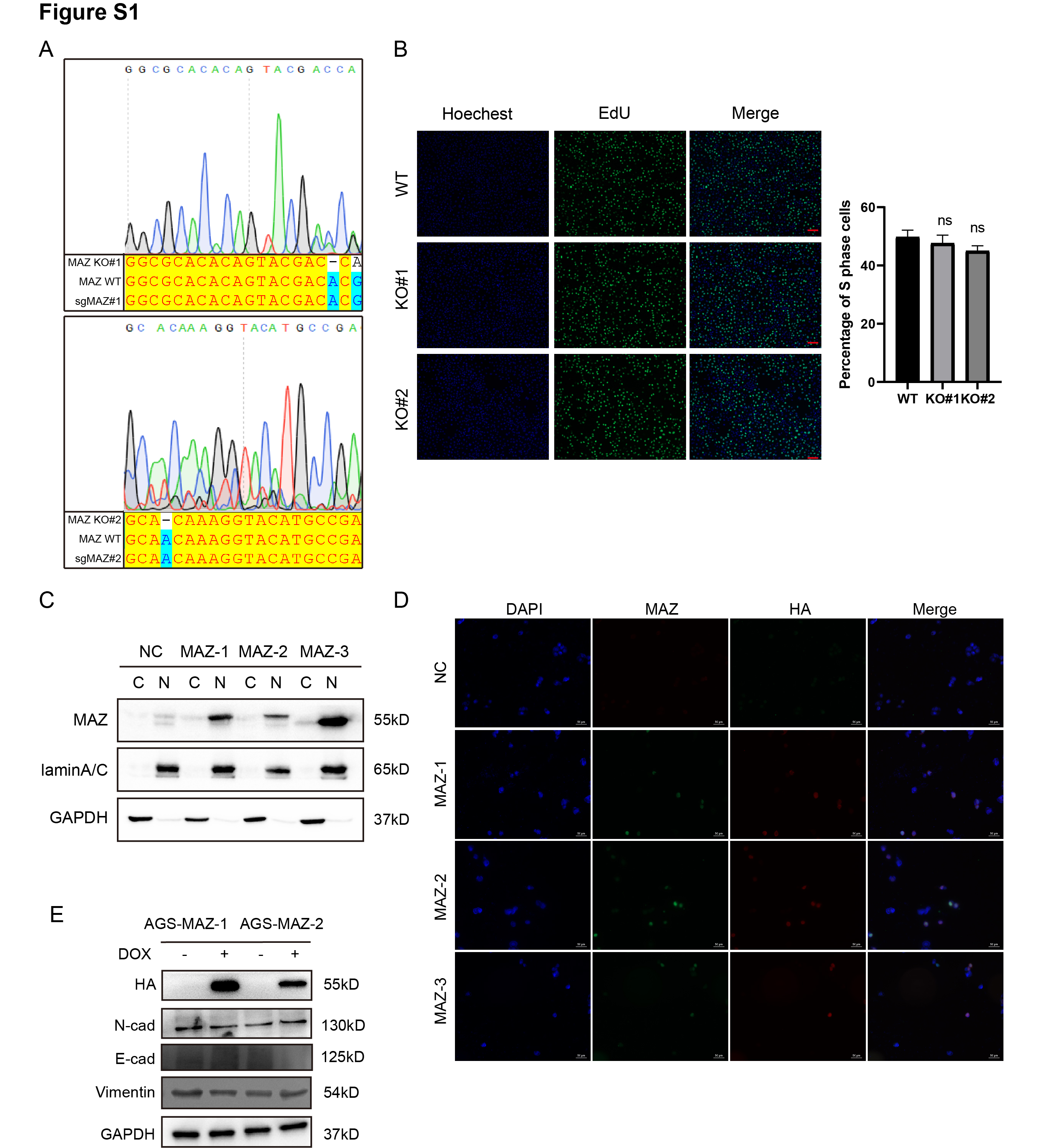

Supplement: Supplementary file 2 — Figure S1. (A) Genomic sequencing analysis of AGS partial knockout cells. (B) The proliferation of cells under MAZ knockout was determined via EdU assays. Scale bar, 100 μm. (C, D) Western blot analysis and immunofluorescence to detect MAZ splice isoform localization in AGS cells expressing MAZ‐1‐HA, MAZ‐2‐HA, or MAZ‐3‐HA. Scale bar, 25 μm. (E) Western blot analysis to the expression of EMT markers in AGS cells expressing MAZ‐1‐HA, and MAZ‐2‐HA. [file CAM4-14-e70977-s003.jpg]
